# Understanding the Associations among Social Vulnerabilities, Indigenous Peoples, and COVID-19 Cases within Canadian Health Regions

**DOI:** 10.3390/ijerph191912409

**Published:** 2022-09-29

**Authors:** Kimberly R. Huyser, Aggie J. Yellow Horse, Katherine A. Collins, Jaimy Fischer, Mary G. Jessome, Emma T. Ronayne, Jonathan C. Lin, Jordan Derkson, Michelle Johnson-Jennings

**Affiliations:** 1 Department of Sociology, The University of British Columbia, Vancouver, BC V6T 1Z1, Canada; 2 School of Social Transformation, Arizona State University, Tempe, AZ 85281, USA; 3 Department of Psychology and Health Studies, University of Saskatchewan, Saskatoon, SK S7N 5A5, Canada; 4 Faculty of Health Sciences, Simon Fraser University, Burnaby, BC V5A 1S6, Canada; 5 School of Public Health and Social Policy, University of Victoria, Victoria, BC V8W 2Y2, Canada; 6 Dalla Lana School of Public Health, University of Toronto, Toronto, ON M5T 3M7, Canada; 7 Director Indigenous Environmental Health and Land-Based Healing Division, School of Public Health, School of Social Work, University of Washington, Seattle, WA 9105, USA

**Keywords:** Indigenous communities, COVID-19 pandemic, social determinants of health, social vulnerability indicators

## Abstract

Indigenous Peoples are at an increased risk for infectious disease, including COVID-19, due to the historically embedded deleterious social determinants of health. Furthermore, structural limitations in Canadian federal government data contribute to the lack of comparative rates of COVID-19 between Indigenous and non-Indigenous people. To make visible Indigenous Peoples’ experiences in the public health discourse in the midst of COVID-19, this paper aims to answer the following interrelated research questions: (1) What are the associations of key social determinants of health and COVID-19 cases among Canadian health regions? and (2) How do these relationships relate to Indigenous communities? As both proximal and distal social determinants of health conjointly contribute to COVID-19 impacts on Indigenous health, this study used a unique dataset assembled from multiple sources to examine the associations among key social determinants of health characteristics and health with a focus on Indigenous Peoples. We highlight key social vulnerabilities that stem from systemic racism and that place Indigenous populations at increased risk for COVID-19. Many Indigenous health issues are rooted in the historical impacts of colonization, and partially invisible due to systemic federal underfunding in Indigenous communities. The Canadian government must invest in collecting accurate, reliable, and disaggregated data on COVID-19 case counts for Indigenous Peoples, as well as in improving Indigenous community infrastructure and services.

## 1. Introduction

The COVID-19 pandemic has posed, and continues to pose, significant and multifaceted threats to population health in Canada and across the globe. In January 2022, almost two years into the pandemic, Canada experienced its largest COVID-19 wave. By August 2022, nearly as many Canadians reportedly died of COVID-19 in 2022 than in 2021 [1]. Although the pandemic has substantially affected populations globally, COVID-19 has unequally impacted individuals and communities based on pre-existing social and economic inequities. Understanding the differential impacts of COVID-19 across Canadian provinces and territories has been further complicated both by the differences between COVID-19 variants and changing provincial reporting practices. Despite these limitations, COVID-19 has had disproportionate impacts on communities of color and lower socioeconomic populations, especially among Indigenous Peoples [2,3,4,5].

Indigenous Peoples in Canada are the original peoples of North America and their descendants. In 2016, more than 1.67 million people in Canada (4.9% of the total population) self-identified as Indigenous. While Indigenous Peoples are monolithically racialized as a collective, Indigenous Peoples are highly diverse with distinct histories and relations to the colonial state. There are more than 630 First Nation communities representing more than 50 Nations and 50 Indigenous languages [6]. While defining Indigenous Peoples is complex with lasting legacies of colonialism and blood quantum politics, the Canadian Constitution officially recognizes three groups of Indigenous Peoples: First Nation (977,230 people, 58.4% of Indigenous Peoples in Canada), Inuit (62,025 people, 3.7%) and Métis (587,545 people, 35.1%).

In addition to the importance of Indigenous Peoples’ health, the focus on Indigenous People’s experiences during the pandemic is particularly important for several reasons. First, Indigenous Peoples in Canada have experienced higher infection and death rates relative to the general Canadian population [2,4,7,8]. Second, as the inequitable outcomes of COVID-19 on marginalized populations are underpinned by pre-existing structural inequities in society (i.e., social vulnerabilities), which have been amplified during the pandemic [4,9,10,11]; Indigenous Peoples experiences highlight additional challenges leading to inequity.

The overall pattern of inequity is clear, but several major limitations undermine our understanding of COVID-19 and Indigenous communities in Canada, namely the incredible diversity of Indigenous Peoples on Turtle Island and the limitations in publicly available COVID-19 data. This lack of accurate and reliable data masks the disaggregated impacts of COVID-19 on the diverse Indigenous populations in Canada—First Nations, Inuit, and Métis [8].

In this paper, we address these limitations by providing a national picture of confirmed COVID-19 cases across provincial health regions in 2020 and 2021, with an emphasis on differential experiences of First Nation, Inuit and Métis peoples. We also examine the association between key social determinants of health and confirmed COVID-19 cases across two time points. Finally, we provide an interpretation of the impact of COVID-19 cases on Indigenous Peoples and the four domains of the Social Vulnerability Index (SVI)—socioeconomic status vulnerability, household composition vulnerability, minority status and language vulnerability, and housing and transportation vulnerability—which provides a framework to understand the relationship between social inequities and COVID-19 cases [12]. The SVI and its categories allow for the identification of place-based vulnerabilities and, in the case of COVID-19, it may allow identification of characteristics of health regions that may be susceptible to illness and disease. Our findings allow more nuanced investigation of COVID-19 effects in Canada in a broader perspective, as well as shed light on the situations of Indigenous Peoples in Canada.

## 2. Materials and Methods

### 2.1. Data

We assembled a unique dataset from multiple sources to examine the associations among key social vulnerability indicators and confirmed COVID-19 cases at two time points: 14 September 2020 (6 months into the pandemic) and 11 November 2021 (20 months into the pandemic). Our dependent variables, COVID-19 cases per health region, came from the Esri Canada Health Regional Archive [13]. Health regions are administrative areas defined by the provincial ministries of health and are the geographic units for which provincial health data are produced [13]. Health region boundaries typically correspond to census geographic units but, in some cases, are delineated by other geographic attributes [13]. Population information and social vulnerability indicators came from the 2016 Canada Census [14]. We also included counts of First Nations communities in each health region; these data were collected from Crown-Indigenous Relations and Northern Affairs Canada [15,16,17,18]. Individual health regions were identified by a unique four-digit numeric code. Since the 2016 Canada Census, the boundaries of some health regions have changed via the merging of two health regions into one. Thus, all data were merged using the 2016 health region code as the common geographic identifier. We calculated the descriptive statistics by assigning weights proportionally by population size of health regions. This unique dataset allowed us to study associations at the health region unit of analysis; in its design, it is an ecological assessment and does not involve individual level correlations, nor is it considered human subject research.

### 2.2. Measures

We created the dependent variables of confirmed COVID-19 cases per 1000 population for both 14 September 2020 and 11 November 2021 in each health region. To create the dependent variables, we divided the cumulative counts of confirmed COVID-19 cases for each date by the health region’s total population then multiplied by 1000. Thus, the dependent variable is the prevalence of COVID-19 cases in health regions at each time point. We log-transformed the dependent variable to meet the parametric requirement for normality for regression analysis, consistent with previous studies on COVID-19 [4,19].

Social conditions and resources, often referred to as the social determinants of health, of a locality and population shape the likelihood of experiencing illness and disease of individuals within a community [19,20]. We conceptualized and measured the social determinants of health using the Social Vulnerability Index (SVI) [12], which provided a framework to understand the relationship between social inequities and COVID-19 cases. The SVI was first developed to quantify the understudied social dimensions of vulnerability to disaster [12], but has been adapted to understand social vulnerability to infectious disease, illness, and Indigenous People’s experiences during the COVID-19 pandemic [3,4,19,21,22,23,24,25,26]. The SVI categorizes social risk factors into four domains: socioeconomic status vulnerability, household composition vulnerability, minority status and language vulnerability, and housing and transportation vulnerability. We included 18 variables from the 4 dimensions of the SVI [27,28]. For the *socioeconomic status vulnerability* dimension, we included four variables: percent of the population living below CAD 35,000, percent of the population who are unemployed, logged median income, and percent of the population without a high school diploma. For the *household composition* vulnerability dimension, we included three variables: percent of the child population aged under 18, percent of elder population aged 65 and older, and percent of single-parent households. For the *minority status and language vulnerability* dimension, we included three variables: percent of visible minority, percent Aboriginal identity—a 2016 Census generated term—(aggregate and by subpopulation), and percent of the population that speak a language other than the two official languages (English and French). For the *housing and transportation vulnerability* dimension, we included five variables: percent of crowded households (i.e., having more than one occupant per room), percent of housing units that are mobile homes, percent of the population who live in subsidized housing, percent of the population whose workers commute more than 60 min, and the percent of the population that commute to work by public transportation.

To capture *COVID-19-related vulnerabilities,* we use the logged population density. Finally, to gauge *historically embedded vulnerability*, we included the number of First Nation communities within a health region as an indicator of the legacy of colonization [29]. We also controlled for the major geographic regions within Canada to account for regional differences: Central Canada, Atlantic Provinces, Prairie Provinces, West Coast, and Northern Territories.

### 2.3. Data Analysis

There were no missing values for COVID-19 cases in provincial health regions during the study period. The final analytic sample included 99 health regions across all of Canada’s provinces and territories. We ran two sets of ordinary least squares (OLS) regressions for each of the two time points: all models included all four dimensions of vulnerability: socioeconomic status vulnerability, housing composition vulnerability, minority status and language vulnerability, and housing and transportation vulnerability, as well as indicators for COVID-19-related vulnerability, historically embedded vulnerability, and census regions. For model 1, we used the aggregate proportion of the population who identify as “Aboriginal identity” as an independent variable, and we stratified model 2 used the disaggregated subpopulations (First Nations, Inuit, Métis, and multiple Aboriginal responses) to look at meaningful heterogenous experiences among these distinct populations. For context, we also created maps showing the location of First Nations communities, COVID-19 prevalence in both time points, and change in COVID-19 prevalence between 2020 and 2021. Analyses occurred in early 2021, and all analyses were conducted using STATA SE 17 (StataCorp, College Station, TX, USA) and ArcGIS Pro (2.8.3) (Esri, Redlands, CA, USA) [30,31].

## 3. Results

Table 1 shows the descriptive statistics of all variables, by total and geographic region. As shown, there are substantial differences in the confirmed COVID-19 cases by geographic region. On average, the rate of confirmed COVID-19 cases per 1000 was 5.4 in September 2020 and 8.5 in November 2021, with variation across geographic regions and the Atlantic provinces and Northern Territories having lower averages in both time points. Table 1 also illustrates the variation across geographic regions by dimensions of socioeconomic status vulnerability and housing composition vulnerability factors. Within the minority status and language vulnerability dimension, there was variability in the percent of the population that identifies as Aboriginal, in that there were higher percentages in the Prairie provinces and Northern Territories. There were more First Nation communities in the West provinces within our historically embedded vulnerability dimension. Along the dimension of housing and transportation vulnerability, Prairie provinces and Northern Territories had larger percentages with crowded households, 4.4 and 9.6, respectively, more than double the total average percentage of 2.4. In terms of logged population density, the Atlantic provinces had the highest mean.

Table 2 presents the results of the OLS regression models to assess the relative impacts of key social vulnerability indicators, historically embedded vulnerability, COVID-19-related vulnerability, and geographic region for September 2020 and November 2021. Within the socioeconomic status vulnerability dimension, the percent of the population who were unemployed had a positive and statistically significant association with COVID-19 cases. Within the household composition vulnerability dimension, in November 2021, there was a negative and statistically significant association between the percent of elders (65 years and older) within the population and COVID-19 cases. Within the minority status and language vulnerability dimensions, we found different associations within the Aboriginal identity subpopulations and COVID-19 cases. At both time points, higher percentage of population that identify as Inuk within a health region had a significant positive association with number of COVID-19 cases. In November 2021, higher percentage of population that identify as Métis within a health region had a significant positive association with number of COVID-19 cases. For the percent of the population who identified as multiple Aboriginal responses, there was a significant negative association with confirmed COVID-19 cases for both 2020 and 2021. For the COVID-19-related vulnerability dimension, the logged population density had a positive and statistically significant association with COVID-19 cases in November 2021. The number of First Nation communities within the historically embedded vulnerability dimension had a statistically significant and negative association in September 2020 and a significant positive association in November 2021. Finally, in the census regions, the Atlantic provinces had negative associations with COVID-19 cases relative to the reference region of Central Canada and the Prairie provinces had a significant positive association with COVID-19 cases.

**Figure 1:**
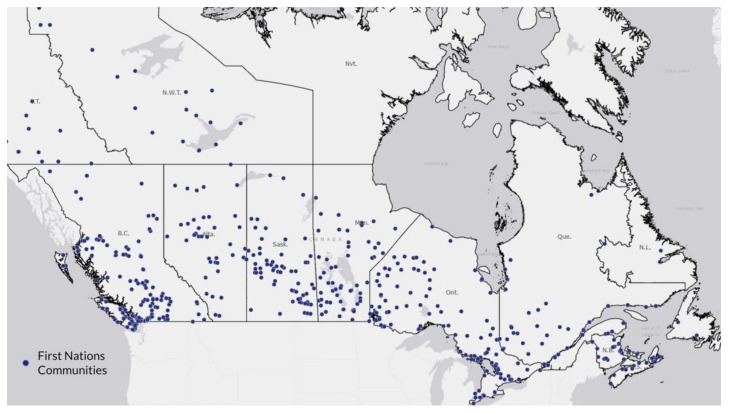
Location of First Nations Communities Across Canada.

**Figure 2:**
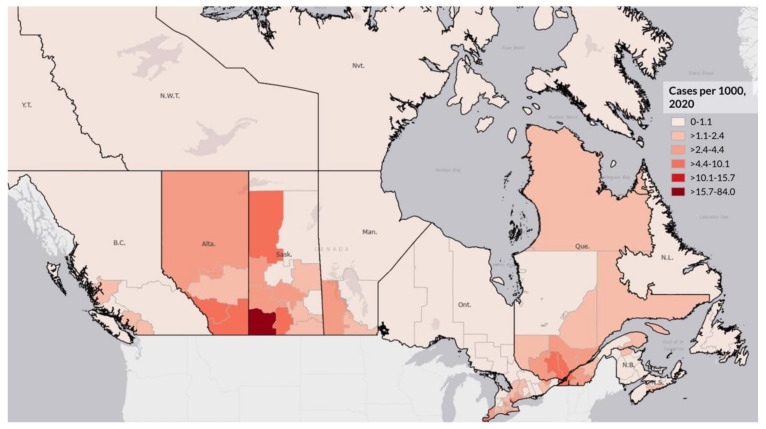
Logged COVID-19 Cases per 1000 in 2020 by Canadian Health Region.

**Figure 3:**
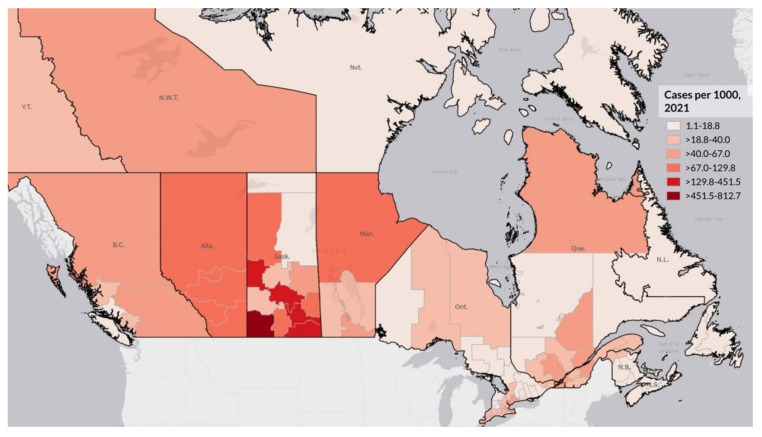
Logged COVID-19 Cases in 2021 per 1000 by Canadian Health Region.

**Figure 4:**
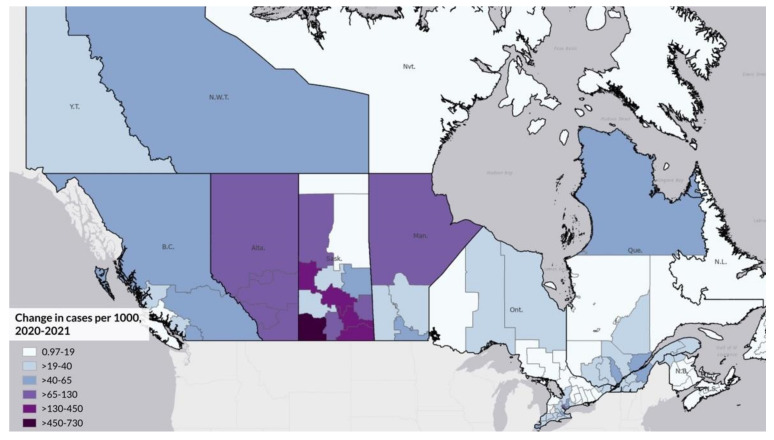
Changes in Logged COVID-19 Cases per 1000 by Canadian Health Region, 2020–2021.

Figure 1 shows the location of First Nation communities across Canada. As shown, there are First Nation communities in the vast majority of provinces and territories, with a higher proportion in the West and Prairie provinces. Figure 2 and Figure 3 illustrate the logged COVID-19 cases per 1000 in 2020 and 2021. In both years, the Prairie provinces had a higher number of COVID-19 cases. Figure 4 shows the changes in logged COVID-19 cases between 2020 and 2021 and many of the provinces saw increases in the presence of COVID-19 across the years. The Prairie provinces continue to have higher case counts of COVID-19.

## 4. Discussion

The ongoing COVID-19 pandemic has continued to pose a significant public health threat to the Canadian population, as well as globally. It has highlighted the complex social vulnerabilities that facilitated the spread of COVID-19. Evidence from the COVID-19 pandemic has suggested that poor and racialized populations have experienced the disproportionate burdens of severe illness and mortality from COVID-19 [2,3,4,5]. Indigenous populations have also experienced substantially higher infection and mortality rates relative to the general Canadian population [2,4,7,8,11,23]. Many on reserve Indigenous communities are remote and have a fragile infrastructure, including limited access to safe housing, clean water, nutrient-dense foods, and transportation [32]. Built-environment conditions can introduce unique challenges in adequately preventing the spread of COVID-19 [11,32,33,34]. There are also gaps in health and social services that impact a large and increasing urban Indigenous population [35]. Due to lack of accurate, reliable, and disaggregated national data on Indigenous populations, there is no national profile of COVID-19 and Indigenous populations. This study sought to fill this gap. It also examined the associations among social determinants of health within a health region and confirmed COVID-19 cases using the SVI dimensions (socioeconomic status vulnerability, household composition vulnerability, minority status and language vulnerability, and housing and transportation vulnerability) and other dimensions relevant to the COVID-19 pandemic. Across most dimensions, we found statistically significant relationships among key variables, such as unemployment and transportation, and COVID-19 cases. This highlights the importance of social determinants of health during the COVID-19 pandemic and perhaps points to opportunities to disrupt distal health mechanisms [21].

The associations found within the *socioeconomic status vulnerability* and *household composition vulnerability* dimensions further extend the literature on the relationship between socioeconomic status and disease to the COVID-19 pandemic [36,37]. We found a positive association between the percent of the population who are unemployed and COVID-19 cases in both 2020 and 2021. This association has been found in other health research between unemployment and poor health outcomes [38]. Addressing this vulnerability is particularly important for Indigenous populations because the unemployment rate amongst Indigenous people was 1.8 times that of non-Indigenous people prior to the pandemic [39,40]. Overall, we did not see a statistical relationship between the household composition variables and COVID-19 cases, except for the percent of the population that is 65 years and older in 2021. Since the beginning of the COVID-19 pandemic, older age has prevailed as the leading risk for both severe illness and death [3]. People over 80 years of age, as of December 2020, accounted for 70% of all COVID-19-related deaths [3]. In our study, we found a negative association between the percent of the elder population and COVID-19 cases in a health region, which is slightly counterintuitive. It may reflect the impact of vaccinations and public health measures that sought to protect the older population in a community. As of July 2021, more than 90 percent of First Nations Elders in British Columbia had received one dose of a COVID-19 vaccine, and more than 84 percent had been fully vaccinated [41]. From a strengths-based lens, these results may also speak to Indigenous community efforts to protect seniors, Elders, and Knowledge Keepers, who are all vital to cultural continuity and protection. Indeed, across urban, rural and remote areas, communities reported working collaboratively to ensure that Elders were protected or isolated from exposure [11]. Across Canada, the 65 years and older population is more likely to be vaccinated than the average adult [42].

Within the *minority status and language vulnerability*, we found differences in the disaggregated categories of Aboriginal identity and COVID-19 cases for both 2020 and 2021, which demonstrates the importance of recognizing the heterogeneity of Indigenous peoples. According to the 2016 Canadian Census, 4.9 percent of the population identify as Indigenous [43]. We found a positive correlation between COVID-19 and Métis and Inuit identity, respectively. Thus, health regions with a higher proportion of the population who identify as either Métis or Inuk were associated with a higher logged number of COVID-19 cases. Indigenous Services Canada provides funds for services and also direct health services to First Nation communities and funds for community health programs for Inuit living in the Inuit Nunangat [44]. Off-reserve First Nations, non-status First Nations, and Métis receive healthcare services from province and territory systems [44]. For Indigenous people who live in rural and remote communities, access to medical care is a major barrier to good health. In 2017, 82% of Inuit in Inuit Nunangat reported that they did not have a family doctor, compared to the rest of the Canadian population, at less than 20% [45]. Indigenous Peoples may also experience barriers in accessing health services due to gaps in service and transportation, and in addition to household composition vulnerabilities, may be spatially marginalized, and experience higher rates of homelessness [11]. More research is needed to understand the diversity of experiences among Indigenous Peoples in Canada, but our work underlines why disaggregated data is essential to both a thorough investigation of the impacts of COVID-19 on Indigenous communities and in relating outcomes to geographic, historic, and community context.

Housing and transportation have been identified as important determinants of health [4,11,23,46,47,48]. We found a negative association between the percent of the population who have tenant households in subsidized housing. It is out of the scope of this study to fully understand the mechanisms that underlie this association, but future research must further investigate the specific mechanisms to better inform policies. We also found a positive association between the percent of workers who commute more than 60 min and COVID-19 cases. Prior research in Canada has found that commuters traveling 60 to 90 min were disproportionately people with physical limitations, visible minorities, low-income workers, those in substandard housing, transit commuters, and those in trades such as mining and agriculture [49]. This finding may indicate exposure burden for those who commute to work and the need for public health measures to lower risks while commuting.

The *COVID-19-related vulnerability, historically embedded vulnerability, and Census regions* of Canada findings highlighted the importance of the characteristics of the geographic area in which people reside and the number of COVID-19 cases. The findings around population density, presence of First Nation communities, and geographic region suggest that historical characteristics and public health mitigation efforts may be related to the number of COVID-19 cases. Throughout the pandemic, Canadian provinces have used various and different approaches to mitigate the spread of COVID-19. The Atlantic provinces of New Brunswick, Nova Scotia, Prince Edward Island, and Newfoundland and Labrador created the Atlantic Bubble in July 2020 wherein the Atlantic provinces all implemented a requirement to isolate for 14 days upon entering one of the provinces from outside of the bubble, whether a resident or not [3]. The Atlantic Bubble allowed residents within the four provinces the freedom of movement and travel without the requirement to self-isolate for cross-provincial travel [3]. The Atlantic Bubble was suspended in November 2020 due to an increase in COVID-19 cases, but was re-implemented in June 2021 following vaccine roll-outs [3,50]. Conversely, many of the Prairie provinces minimized their use of mask mandates and travel restrictions, while making irregular use of COVID-19 dashboards. Saskatchewan, Manitoba, and Alberta have seen the highest rate of cases across Canada since November 2020, surpassing other more populated provinces such as Ontario and Quebec during this time period [51]. Despite all Canadian provinces having a unilateral lockdown in the spring of 2020, Prairie Premiers were hesitant to have a second round of lockdown in the winter of 2020 [51]. In spite of this hesitance, Alberta implemented shutdowns on businesses including casinos, restaurants, and bars starting 13 December 2020 [52] and Saskatchewan followed suit with less severe mandates—shutting down casinos and bingo halls starting December 19th, with other event venues such as arenas, museums and theatres being allowed to operate at reduced capacity [53]. Across the Prairie provinces, there has been a patchwork of governmental recommendations and mandates on masking and vaccination requirements for businesses, schools, and events [54]. For example, Manitoba was the first of the three Prairie provinces to have a province-wide mask mandate on 12 November 2020, with Saskatchewan following on November 19th and Alberta joining in on December 8th [51]. In August 2021, the Premiers and Health Ministers of both Alberta and Saskatchewan stated that they are not eager to enforce public health measures; rather, they are relying on citizens to choose to get a vaccine in order to keep the pandemic at bay [54].

### Strengths and Limitations

The strengths of our study were that we used COVID-19 dashboard counts for two time points in the pandemic, September 2020 and November 2021, and used the 2016 census to construct an innovative health regions level data set (n = 99), which allowed a national view of the relationship between social determinants of health and COVID-19 cases. In addition, this study provided evidence for the relationship between Indigenous Peoples and COVID-19 cases while controlling for SVI dimensions. Furthermore, our approach included a spatial perspective that provided insight into how province and health region characteristics are related to COVID-19. This has the potential of informing place-based policies to mitigate the spatially differing impacts of COVID-19.

We note a few limitations. The study is an ecological assessment at the health region level, and the findings cannot be inferred to individual persons or other units of analysis (i.e., ecological fallacy). The results should be interpreted cautiously because the number of confirmed COVID-19 cases does not reflect the current risk of transmission. It is also a cross-sectional study that captures the associations between social inequalities and the number of confirmed COVID-19 cases based on data collected through September 2020 and November 2021.

## 5. Conclusions

Our study examined the relationship between the social determinants of health and the number of confirmed COVID-19 cases in the 99 health regions across Canada. Our findings emphasize the importance of disaggregated health data on Indigenous populations, as the percentages of individuals who identify as First Nation, Métis, or Inuk (Inuit) have different associations with the number of COVID-19 cases in a health region. Each province and territory had their own approach to mitigating the spread of COVID-19, which differed in effectiveness. In support of this, we found variation in the number of COVID-19 cases across time and geographic region. Our findings also provide further evidence for the influence of social, and historically embedded, risk factors on vulnerability to COVID-19: unemployment, transportation needs, and population density all significantly increase vulnerability to COVID-19.

Through illuminating some of the social determinants of health that are important to COVID-19 case numbers, we provide the opportunity to address the social conditions and the fundamental mechanisms that lead to disease, illness, and vulnerability [21]. Now, countries and societies with Indigenous populations can address the current and historical colonial practices that erode Indigenous health and well-being [55,56]. Providing systemic support for Indigenous Peoples to document and address issues of health and well-being on their own terms will foster sovereignty and self-determination to shape Indigenous futures.

## Figures and Tables

**Table 1:** Descriptive Statistics for Health Regions by National Average and Province/Territory.

	National Average		Central Provinces		Atlantic Provinces		Prairie Provinces		West Provinces		Northern Territories	
	m/%	SD	m/%	SD	m/%	SD	m/%	SD	m/%	SD	m/%	SD
**COVID-19 Cases** ^**a**^												
Logged COVID-19 cases on 14 September 2020	5.4	2.1	6.0	1.9	3.3	1.7	5.3	1.8	6.6	1.4	2.2	0.8
Logged COVID-19 cases on 14 November 2021	8.5	1.8	8.7	1.7	6.5	1.1	9.1	1.3	10.3	0.9	7.1	0.5
**Socio-economic Vulnerability** ^**b**^												
Percent household below CAD 35,000	23.2	5.5	22.5	5.0	27.8	4.6	22.4	6.2	22.8	2.4	17.2	2.0
Percent unemployment	9.6	5.2	7.9	2.4	14.1	4.9	10.4	7.9	7.6	2.3	13.8	6.8
Logged median income	11.1	0.2	11.1	0.2	11.0	0.2	11.2	0.2	11.2	0.1	11.5	0.2
Percent without high school diploma	23.7	9.6	22.1	7.9	24.4	5.7	27.6	13.1	16.8	4.3	31.5	17.6
**Household Composition Vulnerability** ^**b**^												
Percent children under 14	17.6	4.5	16.8	3.5	14.3	1.7	21.4	4.8	15.1	2.5	23.8	7.8
Percent elders 65 and older	17.6	5.0	18.5	4.2	20.8	3.3	14.6	4.9	18.5	4.3	7.8	4.0
Percent single-parent household	17.2	6.1	16.7	4.2	16.4	2.4	18.8	10.4	15.3	1.1	23.3	5.2
**Minority Status and Language Vulnerability** ^**b**^												
Percent visible minority	9.1	12.3	10.0	13.8	3.0	2.5	8.8	8.9	22.4	21.1	6.9	3.8
Percent “aboriginal identity”	14.1	22.7	8.8	18.3	8.1	9.0	26.1	30.1	8.7	7.1	53.3	31.3
First Nations (North American Indian)	8.7	17.4	5.2	13.6	4.4	5.0	19.1	26.5	5.8	5.2	17.2	15.8
Métis	3.1	4.7	1.7	1.8	2.4	2.8	6.8	8.0	2.6	1.8	3.9	4.0
Inuk (Inuit)	2.1	12.4	1.8	12.5	1.0	3.4	0.0	0.0	0.0	0.0	31.8	46.1
Multiple aboriginal responses	0.1	0.1	0.1	0.1	0.1	0.1	0.1	0.1	0.1	0.1	0.3	0.2
Aboriginal not included elsewhere	0.1	0.2	0.1	0.1	0.3	0.5	0.1	0.1	0.1	0.0	0.2	0.1
Percent who speak other than official language	1.0	1.9	1.2	2.3	0.2	0.2	3.0	2.5	2.4	2.8	2.2	3.1
**Housing and Transportation Vulnerability** ^**b**^												
Percent in crowded homes	2.4	4.7	1.7	3.9	0.5	0.4	4.4	6.2	1.8	1.3	9.6	11.0
Percent in mobile homes	2.3	2.6	1.0	2.1	3.4	1.9	3.9	2.5	4.0	3.6	3.8	3.8
Percent in “tenant housing in subsidized housing”	18.7	12.5	22.1	13.2	18.1	5.4	23.4	10.9	12.2	0.8	50.0	30.1
Percent workers commuting more than 60 min	6.9	4.0	8.1	4.8	5.0	1.4	6.0	2.3	7.6	3.7	3.1	0.6
Percent workers commuting by public transportation	4.7	6.6	5.9	7.7	2.4	2.6	3.1	4.3	9.7	9.2	2.0	1.7
**COVID-19-Related Vulnerability** ^**b**^												
Logged population density	2.6	2.5	3.5	2.3	11.0	0.2	1.0	2.2	4.1	3.4	−2.3	3.4
**Historically Embedded Vulnerability** ^**b**^												
Number First Nations communities	8.7	17.4	3.5	6.8	2.0	1.4	7.8	7.6	40.2	16.6	14.3	13.6
**Census Regions** ^**b**^												
Central Canada	0.5	0.5										
Atlantic Provinces	0.2	0.4										
Prairie Provinces	0.2	0.4										
West Coast	0.1	0.2										
Northern Territories	0.0	0.2										

a—Dependent variable. b—SVI domain.

**Table 2:** Regression Results Predicting Logged COVID-19 Cases for 14 September 2020, and 14 November 2021.

	Logged COVID-19 Cases on 14 September 2020		Logged COVID-19 Cases on 14 November 2021	
	Model 1	Model 2	Model 1	Model 2
**Socio-economic Vulnerability** ^**a**^				
Percent household below CAD 35,000	0.06	0.10	0.05	0.10
Percent unemployment	0.11 *	0.12 *	0.07 +	0.06 +
Logged median income	−2.99	−2.70	−2.54	−0.99
Percent without high school diploma	−0.09	−0.10	−0.04	−0.04
**Household Composition Vulnerability** ^**a**^				
Percent children under 14	0.15	0.09	0.08	0.04
Percent elders 65 and older	−0.15	−0.17	−0.17 *	−0.17 *
Percent single-parent household	−0.07	−0.06	−0.02	−0.03
**Minority Status and Language Vulnerability** ^**a**^				
Percent visible minority	0.00	0.03	0.04 +	0.06 *
Percent “aboriginal identity”	−0.05 +		−0.02	
First Nations (North American Indian)		−0.01		−0.01
Métis		0.06		0.12 *
Inuk (Inuit)		0.30 *		0.16 +
Multiple aboriginal responses		−8.52 *		−8.56 **
Aboriginal not included elsewhere		−0.38		0.13
Percent who speak other than official language	−0.06	−0.26	−0.32	−0.53 **
**Housing and Transportation Vulnerability** ^**a**^				
Percent in crowded homes	0.12	0.01	−0.02	−0.03
Percent in mobile homes	0.07	0.10	0.04	0.06
Percent in “tenant housing in subsidized housing”	−0.06	−0.06 *	−0.04 *	−0.05 *
Percent workers commuting more than 60 min	0.09 *	0.10 *	0.06 *	0.05 +
Percent workers commuting by public transportation	0.05	0.03	0.01	0.00
**COVID−19-Related Vulnerability** ^**a**^				
Logged population density	0.13	0.15	0.30 **	0.29 **
**Historically Embedded Vulnerability** ^**a**^				
Number First Nations communities	−0.01	−0.01 *	0.03 *	0.03 *
**Census Regions** ^**a**^				
Atlantic Provinces	−2.49 ***	−2.75 ***	−1.91 ***	−1.98 ***
Prairie Provinces	0.05	0.15	0.99 **	0.74 *
West Coast	0.22	0.28	0.04	0.23
Northern Territories	−1.84	0.53	−0.31	2.03 +
Constant	40.13	37.71	37.44	20.44
R-squared	0.76	0.78	0.83	0.85

a—SVI domain, + *p* < 0.1, * *p* < 0.05, ** *p* < 0.01, *** *p* < 0.0001.

## Data Availability

Publicly available datasets were analyzed in this study. This data can be found here: https://resources-COVID19canada.hub.arcgis.com/maps/health-regional-archive-public-view/about and Statistics Canada Census Profile, 2016 Census.
